# Acupuncture treatment for lumbar disc herniation with scoliosis: A case report

**DOI:** 10.1097/MD.0000000000036684

**Published:** 2023-12-29

**Authors:** Qing Liu, Han Zhang, FangXuan Lin, LiFang Chen, ZhangLian Wang

**Affiliations:** a The Third College of Clinical Medicine, Zhengjiang Chinese Medical University, Hangzhou, China; b Department of Acupuncture and Moxibustion, The Third Affiliated Hospital of Zhejiang Chinese Medical University, Hangzhou, China.

**Keywords:** acupuncture, case report, lumbar disc herniation with scoliosis

## Abstract

**Introduction:**

Lumbar disc herniation (LDH) with scoliosis usually refers to lumbar disc herniation caused by scoliosis, which is a postural compensatory deformity to reduce low back and leg pain, mostly with nonstructural changes. Scoliosis may disappear after the treatment of LDH.

**Patient concerns:**

At present, this kind of scoliosis is mainly treated with medicine and surgery, but all these methods may have some adverse effects.

**Diagnosis:**

A 24-year-old female patient was admitted to the acupuncture department of our hospital due to unbearable pain caused by LDH.

**Interventions:**

According to the patient condition, the acupuncture treatment plan was adopted by Professor Wang Zhanglian, a famous Chinese medicine practitioner.

**Outcomes:**

After 12 weeks of acupuncture treatment, the patient low back pain was significantly relieved.

**Conclusion:**

This case suggests that acupuncture may be an effective alternative treatment for LDH.

## 1. Introduction

Lumbar disc herniation (LDH) is a common and prevalent clinical condition, and the number of patients has increased in recent years. Some patients with LDH may have scoliosis at the same time, which affects the mechanical joint structure of the spine and trunk and can seriously affect their quality of life. LDH with scoliosis is clinically treated with a stepwise approach, from conservative treatment, minimally invasive surgery, conventional decompression surgery, non-fusion fixation surgery, to fusion fixation surgery. 80% to 90% of patients can be cured or improved with non-surgical treatment.^[1]^ Therefore, conservative treatment is the treatment of choice for LDH, which includes anti-inflammatory and pain relievers, neurotropics, Traditional Chinese Medicine (TCM), acupuncture and massage, physical therapy, and life coaching.^[2,3]^

Acupuncture is a common clinical treatment for LDH. International researches has found extensive clinical improvements in LDH patients with acupuncture by using the Visual Analogue Scale/Score (VAS), The Oswestry Disability Index (ODI) and Short form-36 quality-of-life health survey scores (SF-36QOL) at long-term 5-year follow-up.^[4]^ Domestic studies have also shown that the use of acupuncture to treat patients with LDH can achieve good short and long-term results, and can reduce the level of lumbar pain and inflammatory factors, improving the function of the lumbar spine and the quality of life of patients. The mechanism is that acupuncture can promote the metabolism of the nerve itself, reduce the inflammatory response of the nerve and its surrounding tissues, improve the blood microcirculation of the perineural tissues, and affect the transmission of neuroanalgesic transmitters, thus achieving therapeutic effects.^[5]^

Acupuncture has the advantages of safety, low cost and quick results, but also has the disadvantages of being prone to recurring diseases and requiring multiple treatments. Professor Wang Zhanglian treatment of LDH emphasizes the clarification of the etiology of the disease, the flexible selection of treatment methods and the phased treatment; and on the other hand, the importance of identifying the meridians and selecting empirical acupoints for treatment. Compared with traditional acupuncture for LDH, it has a more stable treatment effect.

## 2. Case presentation

**Figure 1.**
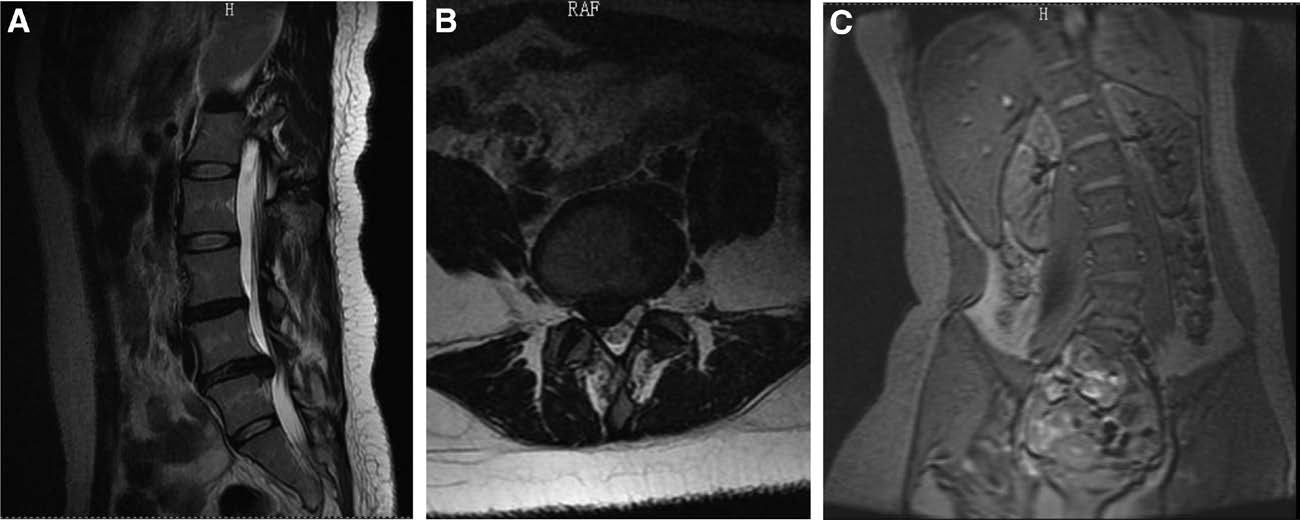
On May 4, 2017, the patient complained of recurrent right-sided lower extremity reflex pain with activity limitation and lumbar pain for more than 3 months. An MRI examination was performed at the local hospital, and the MRI scan of the lumbar spine suggested: L4/5 disc degeneration and bulging; L5/S1 disc right posterior prolapse with right nerve root compression. Mild degeneration of the lumbar spine, left lateral curvature, and lumbarization of the sacral spine with endplate inflammation at the superior margin of S1 considered (Fig. 1A–C).

A female patient, 24 years old, was diagnosed with lumbar L5/S1 disc herniation with left lumbar curvature. For the past 2 years, the patient has been feeling soreness and numbness in her right leg. On May 4, 2017, the patient complained of recurrent right-sided lower extremity reflex pain with activity limitation and lumbar pain for more than 3 months. An MRI examination was performed at the local hospital, and the MRI scan of the lumbar spine suggested: L4/5 disc degeneration and bulging; L5/S1 disc right posterior prolapse with right nerve root compression. Mild degeneration of the lumbar spine, left lateral curvature, and lumbarization of the sacral spine with endplate inflammation at the superior margin of S1 are considered (Fig. 1A–C). The patient was diagnosed with lumbar L5/S1 disc herniation with lumbar left-sided curvature. The patient back pain was unbearable, and the doctor recommended surgery as soon as possible, but the patient refused surgery based on her fear of surgery and began to seek acupuncture treatment.

## 3. Acupuncture and moxibustion treatment

**Figure 2.**
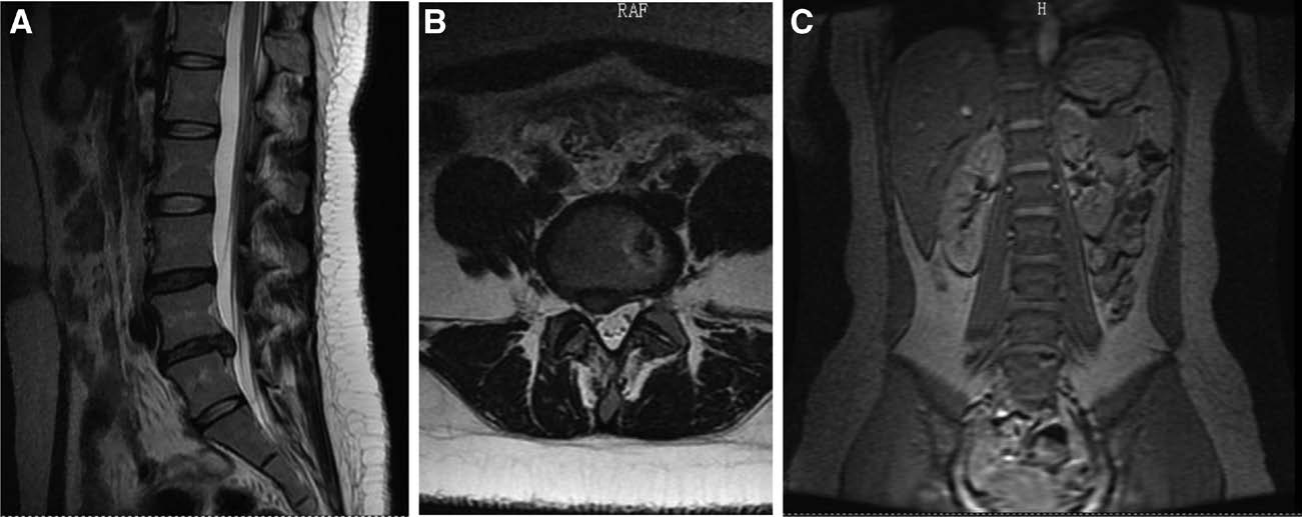
The selected acupoints are as follows: Shenshu (BL23), Qihaishu (BL24), Dachangshu (BL25), Guanyuanshu (BL26), Huantiao (GB30), Huanzhong (Extra35), Chengfu (BL36), Yinmen (BL37), Weizhong (BL40), Chengshan (BL57), Kunlun (BL60). Above selected acupoints are used on both sides (Fig. 4).

Prior to acupuncture treatment, we fully understood the patient condition. The patient currently has unbearable lumbar pain and right lower limb reflex pain with limited movement. Combining the patient condition, Professor Wang Zhanglian adopted his empirical treatment method. The selected acupoints are as follows: Shenshu (BL23), Qihaishu (BL24), Dachangshu (BL25), Guanyuanshu (BL26), Huantiao (GB30), Huanzhong (Extra35), Chengfu (BL36), Yinmen (BL37), Weizhong (BL40), Chengshan (BL57), Kunlun (BL60). Above selected acupoints are used on both sides. (Fig. 2). The specific operation steps are as follows: firstly, we disinfected the acupoints locally and then quickly inserted 0.25 × 40 mm disposable acupuncture needles (Huatuo brand, manufactured by Su Hou Medical Devices, Suzhou, Jiangsu Province, China) into the skin at a certain depth. The patient was placed in the prone position, and 0.30 × 50 mm disposable milli-needle was used to pierce 30 to 40 mm depth directly in acupoints of Shenshu, Qihaishu, Dachangshu, and Guanyuanshu; 0.35 × 0.75 mm disposable milli-needle was used to pierce 30 to 45 mm depth directly in acupoints of Huantiao and Huanzhong; 0.30 × 50 mm disposable milli-needle was used to pierce 30 to 40 mm depth directly in acupoints of Chengfu, Yinmen, Weizhong, Chengshan, and Kunlun, and all acupoints were required to obtain Qi, and the needles were retained for 30 minutes after obtaining Qi (Table 1). The treatment method took 3 times a week for 12 weeks.

**Table 1:** Acupuncture point information table.

Acupoint	Position	Acupuncture depth
BL23 Shenshu	Under the spinous process of the second lumbar spine, 1.5 **inches** apart, between the lumbar dorsal fascia, the longissimus muscle and the iliocostalis	30–40 mm
BL24 Qihai shu	Under the spinous process of the third lumbar spine, with a lateral opening of 1.5 inches	30–40 mm
BL25 Dachangshu	Under the spinous process of the 4th lumbar spine, with a lateral opening of 1.5 inches	30–40 mm
BL26 Guan Yuanshu	Under the spinous process of the 5th lumbar spine, with a lateral opening of 1.5 inches	30–40 mm
GB30 Huantiao	At the intersection of the outer 1/3 and middle 1/3 of the line connecting the highest point of the greater trochanter of the femur and the sacral hiatus	30–45 mm
EX-LE1 Huanzhong	The midpoint of the connection between the Huantiao and the waist shu	30–45 mm
BL36 Chengfu	Behind the thighs, at the midpoint of the horizontal lines below the buttocks	30–40 mm
BL37 Yinmen	At the back of the thighs, when the Chengfu is connected to the center of the Weizhong, the Chengfu is lowered by 6 inches	30–40 mm
BL40 Weizhong	Located in the posterior knee area, at the midpoint of the popliteal striation	30–40 mm
BL57 Chengshan	In the middle of the back of the lower leg, between Weizhong and Kunlun, there is a sharp angle depression under the abdomen of the gastrocnemius muscle	30–40 mm
BL60 Kunlun	Behind the outer ankle of the foot, the depression between the tip of the outer ankle and the Achilles tendon	30–40 mm

## 4. Clinical results

**Figure 3.**
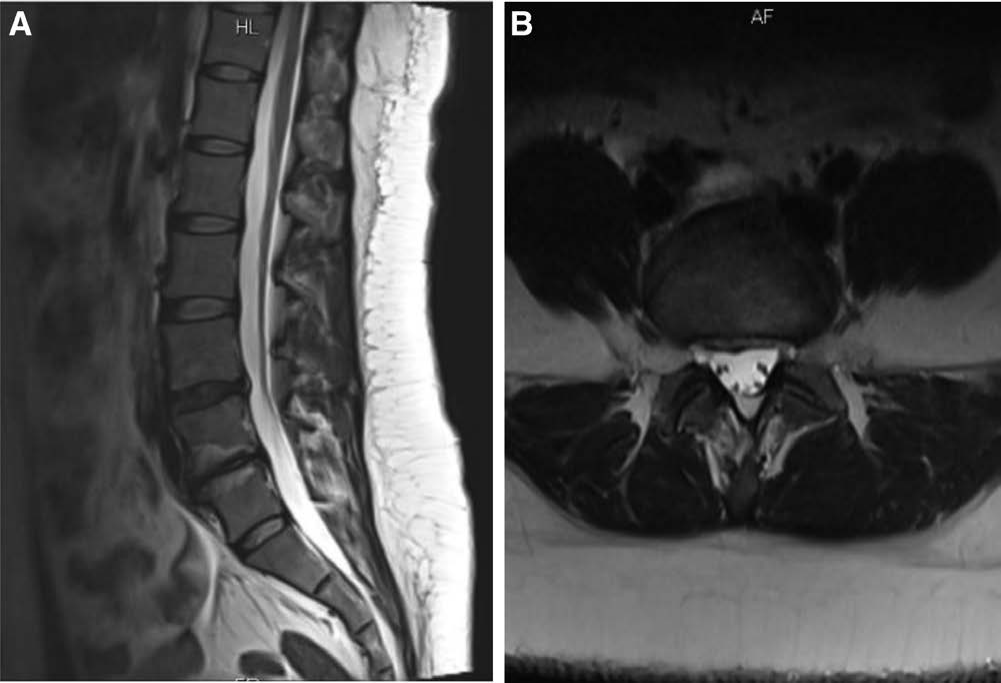
After 12 weeks of treatment, the patient symptoms basically disappeared, and the lumbar MRI scan was reviewed on November 17, 2017: degenerative bulging of the L4/5 disc; degenerative and prolapsed L5/S1 disc; mild lumbar degeneration; sacral lumbarization; L4/5 adjacent vertebral terminal inflammation (Fig. 2A–C).

**Figure 4.**
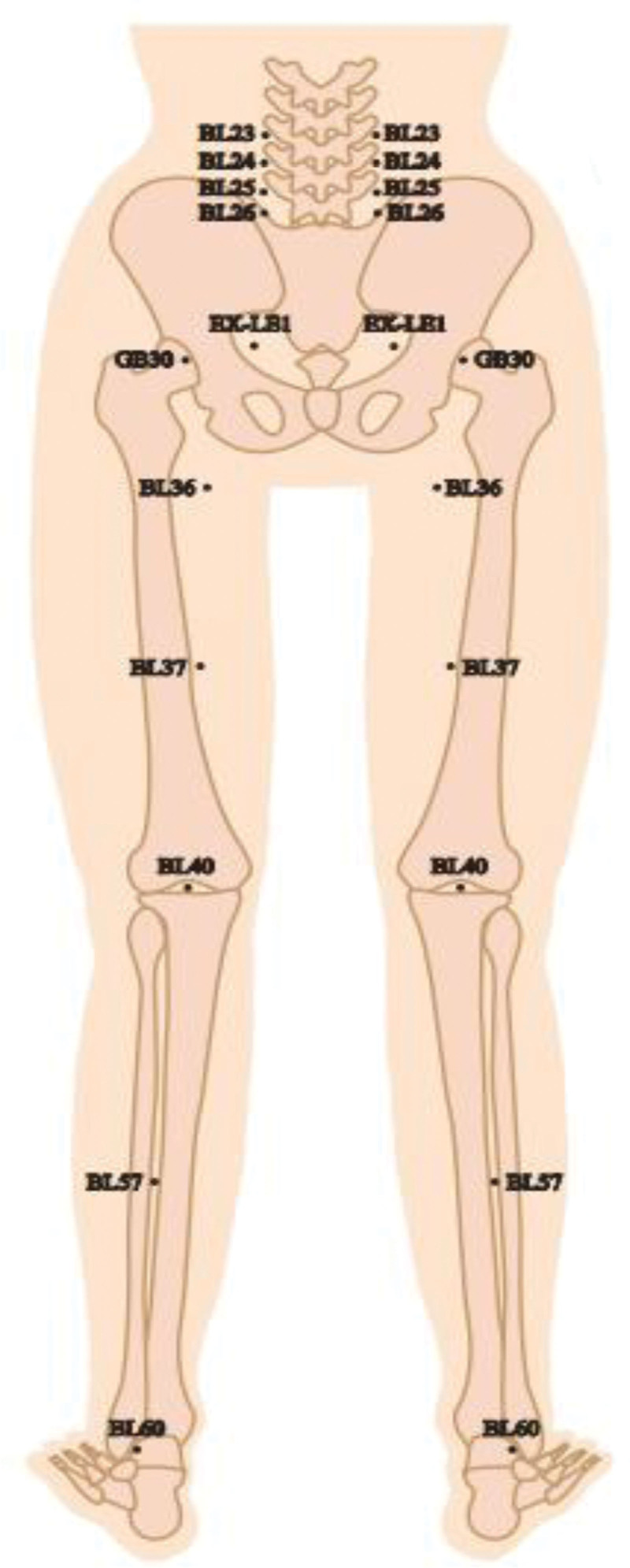
5 years after follow-up, the patient lumbar pain and lower extremity pain did not recur, and the lumbar MRI scan was reviewed on October 11, 2022: lumbar degeneration, degenerative bulging of L4/5 and L5/S1 discs, and endplate inflammation of L5 and S1 vertebrae (Fig. 3A,B).

After 2 weeks of acupuncture treatment, the patient showed significant improvement in lumbar pain and relief of reflex pain in the right lower limb, still with restricted movement. After 12 weeks of treatment, the patient symptoms basically disappeared, and the lumbar MRI scan was reviewed on November 17, 2017: degenerative bulging of the L4/5 disc; degenerative and prolapsed L5/S1 disc; mild lumbar degeneration, sacral lumbarization, L4/5 adjacent vertebral terminal inflammation (Fig. 3A–C). 5 years after follow-up, the patient lumbar pain and lower extremity pain did not recur, and the lumbar MRI scan was reviewed on October 11, 2022: lumbar degeneration, degenerative bulging of L4/5 and L5/S1 discs, and endplate inflammation of L5 and S1 vertebrae (Fig. 4A,B).

## 5. Discussion

LDH is caused by degeneration and injury of the lumbar disc, leading to rupture of the annulus fibrosus and destabilization of the internal and external mechanical balance of the spine, causing the nucleus pulposus to protrude and compress the nerve roots and cauda equina. It belongs to the category of “low back pain” in TCM. External exertional injuries and falls, stagnation of qi and blood channels, damage to the orifice and marrow, protruding from the orifice, obstructing the spinal cord, stagnation of qi and blood along the meridians, resulting in pain and numbness in the Sun, Yangming, and Shaoyang meridians.

Scoliosis is a continuous deviation of multiple vertebrae in the coronal plane of the spine from the midline of the spine to form a lateral bend, as well as an abnormal sequence in the sagittal or axial plane, resulting in a forced imbalance in the spinal stage.^[6,7]^ It can be divided into primary and secondary scoliosis. Scoliosis combined with LDH usually belongs to the secondary. Compensatory lateral bending of the lumbar spine can enlarge the nerve root canal diameter on the convex side and thus reduce the compression symptoms of the herniated nucleus pulposus.^[8]^ The spatial location of the protruding nucleus pulposus and the nerve root determines the direction of scoliosis. If the nucleus pulposus protrudes in front of the nerve root, the spine bends laterally to the healthy side, which is lateral convexity to the affected side; if the nucleus pulposus protrudes in front of the nerve root, the spine bends laterally to the affected side, which is lateral convexity to the healthy side.^[9]^ As the disease progresses and increases the rotation of the spine in the axial position, progressive subluxation of the disc in the sublateral junction area occurs, aggravating the misalignment of the posterior lumbar joints.

According to the medical history, clinical symptoms, and signs of disc herniation, combined with the patient imaging data, the current treatment methods mainly include conservative treatment and surgical treatment. For patients with a medical history of <6 months and no progressive neurological dysfunction, conservative treatment is often preferred, such as medication, physical therapy, acupuncture, TCM treatment, etc. The patient in this case had a 3-month interval between onset and consultation, no significant muscle weakness, and no cauda equina symptoms, so conservative treatment was chosen. At present, domestic and international guidelines recommend anti-inflammatory analgesics as the first-line medication for the treatment of disc herniation, and good symptomatic treatment results have been achieved. However, anti-inflammatory analgesics carry a potential risk of gastrointestinal adverse reactions and need to be used with caution or together with gastroprotective drugs for patients with prior GI disease, increasing the financial burden on patients and decreasing compliance. Other drug such as myorelaxants and neurotrophic drugs, are often used in combination with anti-inflammatory analgesics and are not effective alone. In addition, there is still a high risk of symptom recurrence in patients who are effectively treated with medication. TCM has accumulated rich clinical experience in the treatment of disc herniation and is widely recognized and accepted. Nevertheless, TCM also suffers from gastrointestinal and other adverse effects, decoction is difficult, and there is a risk of recurrence for patients who are effectively treated.

Acupuncture is the use of metal needles stabbed into the human body acupoints, which stimulates the meridian points of the bladder meridian on both sides to unblock the qi and blood, relax the tendons, and loosen the adhesions, thus causing local inflammation and edema to dissipate and stop the pain. Acupuncture changes the relative position of the nerve root and the protrusion, relieves or releases the compression of the protrusion, and reduces or eliminates the pain. In this case, the patient symptoms improved significantly 2 weeks after receiving acupuncture treatment. At the end of the 12-week treatment, the symptoms had largely disappeared, indicating that acupuncture was effective. After up to 5 years of follow-up, there was no recurrence of the symptoms of the herniated disc. Repeated MRI data showed the disappearance of the herniation, indicating the lasting and stable effect of acupuncture treatment. Moreover, during and after each treatment, the patient had no obvious complaints of discomfort and had a good medical experience.

LDH causing tilt and collapse of the intervertebral space, rotation, and displacement of the vertebral body, leading to structural mechanical and morphological pathological changes in the lumbar spine, is also the main cause of secondary scoliosis. The formation of scoliosis produces compressive stresses on the concave side of the scoliotic spine and tensile stresses on the convex side. The 2 types of stresses, respectively cause changes in the pattern of growth, maturation, and degeneration of the chondrocytes on both sides of the vertebral body, resulting in the aggravation of scoliosis. Therefore, changing the asymmetric stress on both sides of the spine is the key to the treatment of scoliosis.^[10]^ The treatment of acupuncture focuses on adjusting the direction of the 4 forces of the bilateral psoas major and bilateral erector spinae muscles, restoring their isometric contrac function, and balancing the stresses on both sides of the scoliosis in order to improve the lumbar curvature as well as the scoliosis. Acupuncture treatment of LDH combined with scoliosis can reduce pain and improve the scoliosis Cobb angle and lumbar curvature arc. In this case, after 12 weeks of acupuncture treatment, the scoliosis was significantly improved and the spinal force line was corrected. No recurrence of scoliosis was found during the 5-year follow-up in this case.

## Author contributions

**Conceptualization:** Fangxuan Lin.

**Formal analysis:** LiFang Chen.

**Methodology:** Zhanglian Wang.

**Writing – original draft:** Qing Liu.

**Writing – review & editing:** Han Zhang.
